# Pure fruit juice and fruit consumption and the risk of CVD: the European Prospective Investigation into Cancer and Nutrition–Netherlands (EPIC-NL) study

**DOI:** 10.1017/S0007114518003380

**Published:** 2018-12-18

**Authors:** Floor R. Scheffers, Jolanda M. A. Boer, W. M. Monique Verschuren, Martijn Verheus, Yvonne T. van der Schouw, Ivonne Sluijs, Henriëtte A. Smit, Alet H. Wijga

**Affiliations:** 1 Center for Nutrition, Prevention and Health Services, National Institute for Public Health and the Environment (RIVM), PO Box 1, 3720 BA Bilthoven, The Netherlands; 2 Julius Center for Health Sciences and Primary Care, University Medical Center Utrecht, Utrecht University, PO Box 85500, 3508 GA Utrecht, The Netherlands; 3 The Hague University of Applied Sciences, PO Box 13336, 2501 EH The Hague, The Netherlands

**Keywords:** Pure fruit juice, Fruit, CVD, CHD, Stroke, Dietary guidelines, European Prospective Investigation into Cancer and Nutrition–Netherlands

## Abstract

Dietary guidelines for pure fruit juice consumption differ between countries, regarding the question whether pure fruit juice is an acceptable alternative for fruit. Currently, little is known about pure fruit juice consumption and the risk of CVD. In this prospective cohort study, we studied the association of pure fruit juice and fruit consumption with the incidence of fatal and non-fatal CVD, CHD and stroke and investigated the differences in association with pure fruit juice consumption between low and high fruit consumers. A validated FFQ was used to estimate dietary intake of 34 560 participants (26·0 % men and 74·0 % women) aged 20–69 years from the European Prospective Investigation into Cancer and Nutrition–Netherlands study. Adjusted hazard ratios (HR) were estimated using Cox regression after average follow-up of 14·6 years. Compared with no consumption, pure fruit juice consumption up to 7 glasses/week – but not consumption of ≥8 glasses – was significantly associated with reduced risk of CVD and CHD, with HR from 0·83 (95 % CI 0·73, 0·95) to 0·88 (95 % CI 0·80, 0·97). Consumption of 1–4 and 4–8 glasses/week was significantly associated with lower risk of stroke with HR of 0·80 (95 % CI 0·64, 0·99) and 0·76 (95 % CI 0·61, 0·94), respectively. Associations did not differ considerably between low and high fruit consumers. The highest three quintiles of fruit consumption (≥121 g/d) were significantly associated with lower incidence of CVD, with HR of 0·87 (95 % CI 0·78, 0·97) and 0·88 (95 % CI 0·80, 0·98). In conclusion, although we observed favourable associations of moderate pure fruit juice consumption with CVD, for now consumption of whole fruit should be preferred because the evidence of the health benefits of fruit is more conclusive.

Although the inverse association between fruit consumption and the risk of CVD is well established in epidemiological studies^(^ ^1^ ^–^ ^4^ ^)^, epidemiological evidence on the association between pure fruit juice consumption and the risk of CVD morbidity and mortality is limited and inconsistent^(^ ^5^ ^–^ ^7^ ^)^. Pure fruit juice is defined as 100 % fruit juice without added sugars or artificial sweeteners and other components as artificial colours or preservatives. Pure fruit juice can be both fresh juice and bottled juice from concentrate. The 2015–2020 Dietary Guidelines for Americans consider pure fruit juice nutritionally similar to whole fruit and state that half of the recommended daily fruit intake may be replaced by pure fruit juice^(^ ^8^ ^)^. In line with this, dietary guidelines from the UK, updated in 2016, state that pure fruit juice can contribute one portion to the ‘5 a day’ target but should be restricted to a portion size of 150 ml/d^(^ ^9^ ^)^. Replacing fruit by pure fruit juice might be a practical solution for people to meet the recommendation for fruit consumption when for any reason more fruit consumption is difficult. In contrast, in the Dutch dietary guidelines of 2015^(^ ^10^ ^)^ pure fruit juice is classified in the same category as ‘sugar-containing beverages’ because of its comparable sugar content. Therefore, the advice is to keep consumption of pure fruit juice to a minimum.

Pure fruit juice contains less dietary fibre and vitamin C compared with whole fruits. However, pure fruit juice still contains a high concentration of polyphenols^(^ ^11^ ^,^ ^12^ ^)^, which might reduce the risk of CVD^(^ ^13^ ^–^ ^16^ ^)^. Most research on pure fruit juice is limited to cardiometabolic risk factors for CVD, such as blood pressure and serum cholesterol. A meta-analysis of nineteen randomised controlled trials showed that pure fruit juice consumption significantly lowered diastolic blood pressure by an average of 2 mmHg^(^ ^17^ ^)^. No effect was seen on total cholesterol (TC), HDL-cholesterol and LDL-cholesterol. The conflicting dietary guidelines emphasise the importance of more research on the health benefits or adverse effects of pure fruit juice consumption. In addition, knowledge about the benefit of pure fruit juice consumption for people with a low fruit intake could be important for unambiguous dietary guidelines for replacing fruit with pure fruit juice. Therefore, the aim of this study is to investigate the association of pure fruit juice and fruit consumption with CVD in the European Prospective Investigation into Cancer and Nutrition–Netherlands (EPIC-NL) cohort and to assess the association between pure fruit juice consumption and CVD for low and high fruit consumers.

## Methods

### Study population

The EPIC-NL study is the Dutch contribution to the EPIC. The EPIC-NL study consists of two cohorts: the Monitoring Project on Chronic Disease Risk Factors (MORGEN) cohort and the Prospect cohort. The MORGEN cohort consists of 22 654 men and women aged 20–65 years randomly selected from the Dutch population in three towns in the Netherlands (Amsterdam, Doetinchem and Maastricht). The Prospect cohort consists of 17 357 women aged 50–69 years, from the Dutch town of Utrecht or its vicinity, who participated in a breast cancer screening programme. For both cohorts, participants were recruited between 1993 and 1997. The study complies with the Declaration of Helsinki and was approved by the institutional board of the University Medical Center Utrecht (Prospect) and the Medical Ethical Committee of TNO Nutrition and Food Research (MORGEN). All participants gave written informed consent. A detailed description of the study design has been published previously^(^ ^18^ ^)^.

**Fig. 1:**
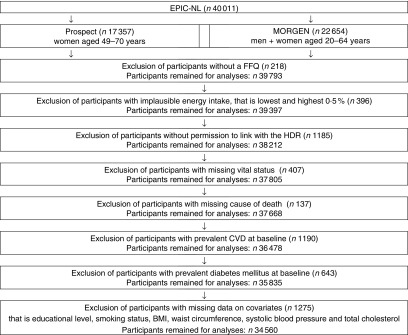
Flowchart of participants excluded from the study. EPIC-NL, European Prospective Investigation into Cancer and Nutrition–Netherlands; MORGEN, Monitoring Project on Chronic Disease Risk Factors; HDR, hospital discharge register.

Exclusion criteria for the present study were missing FFQ (*n* 218), extremely low or high reported energy intake (i.e. those in the lowest or highest 0·5 % of the ratio of energy intake over basal metabolic rate) (*n* 396), no permission to link with the Dutch Hospital Discharge Diagnosis Database (*n* 1185), missing vital status (*n* 407), missing cause of death (*n* 137), prevalent CVD at baseline based on self-report or identified through linkage with the Dutch Hospital Discharge Diagnosis Database (*n* 1190), prevalent diabetes mellitus at baseline based on self-report (*n* 643) or missing data on covariates, that is, educational level, smoking status, BMI, waist circumference, systolic blood pressure and TC (*n* 1275). After exclusion, 34 560 participants remained for the analyses (Fig. 1).

### Exposure assessment

Dietary intake was obtained from a self-administered FFQ containing questions on the average consumption of 178 food items during the year preceding enrolment. The FFQ has been validated for fruit consumption against twelve 24 h recalls. Spearman’s correlation coefficients were 0·68 in men and 0·56 in women^(^ ^19^ ^,^ ^20^ ^)^. Participants indicated their consumption of fruit and pure fruit juice in portions or glasses per day, per week, per month or per year, or as never, for each item separately. For fruit, a portion size of 100 g was used. For fruit juice, we used the standard portion size of 150 ml.

### Outcome assessment

Data on morbidity were obtained from the national hospital discharge register, covering all general and university hospitals and most specialised hospitals in the Netherlands. These data were linked to the EPIC-NL cohort based on information on date of birth, postal code, sex and general practitioner, using a validated probabilistic method^(^ ^18^ ^)^. Data on morbidity were coded according to the International Classification of Diseases (ICD)-9. Data on vital status were obtained from the municipal population register. For those who died, data on the cause of death were obtained from Statistics Netherlands. Data until 1996 were coded according to ICD-9 and data after 1996 were coded according to ICD-10. Follow-up was completed until 31 December 2010. Outcomes for the present study were CVD, CHD and stroke, either fatal or non-fatal. The first event was used as the outcome. CVD was coded according to ICD-9 as 410–414, 427.5, 798.1, 798.2 and 798.9 (CHD), 415.1 (pulmonary embolism and infarction), 428 (heart failure), 443.9 (peripheral vascular disease, unspecified), 430–438 (cerebrovascular event), 440–442 (atherosclerosis, aortic aneurysm and dissection, other aneurysm) and 444 (arterial embolism and thrombosis). According to ICD-10, CVD was coded as I20–I26, I46 and R96 (CHD), I60–I67, I69, G45 (cerebrovascular event), I70–I74 (atherosclerosis, aortic aneurysm and dissection, other aneurysm, other peripheral vascular diseases and arterial embolism and thrombosis) and I50 (heart failure). CHD was coded as 410–414 according to ICD-9 and as I20–I25 according to ICD-10. Stroke was coded as 430–434 and 436 according to ICD-9 and as I60–66 according to ICD-10.

### Covariates

Educational level was defined as low (primary education, lower vocational education, advanced elementary education), intermediate (intermediate vocational education, completion of first 3 years of higher general secondary education) and high (completed higher general secondary education, higher vocational education and university). Cigarette, cigar or pipe smoking was classified as current, former or never. Physical activity was assessed using the validated^(^ ^21^ ^)^ EPIC physical activity questionnaire and classified according to the Cambridge physical activity index into (moderately) active and (moderately) inactive^(^ ^22^ ^)^. Physical activity was not assessed with the EPIC questionnaire in the first year (1993) of the MORGEN study. Therefore, for 14 % of the EPIC-NL cohort, missing values were imputed using single imputation (SPSS Missing Value Analysis procedure)^(^ ^23^ ^)^. The Dutch Healthy Diet index 2015 (DHD15-index) was used as indicator for dietary habits. This index is based on the adherence to the Dutch dietary guidelines of 2015^(^ ^10^ ^)^ and consists of the following fifteen components: vegetables, fruit, wholegrain products, legumes, nuts, dairy products, fish, tea, fats and oils, filtered coffee, red meat, processed meat, sugar-sweetened beverages, alcohol and salt. For all these components, a score between 0 (no adherence) and 10 (complete adherence) is assigned, which results in a total score ranging from 0 to 150 points^(^ ^24^ ^)^. In the EPIC-FFQ, no distinction was made between types of coffee (filtered or unfiltered). Therefore, the component score for coffee was not included in the DHD15-index we used. Furthermore, pure fruit juice (part of the component sugar-sweetened beverages) and fruit were also not included in the DHD15-index because these were exposure variables in our study. Consequently, our DHD15-index was the sum of thirteen components ranging from 0 (no adherence) to 130 (complete adherence). Waist circumference, height and weight were measured according to the standardised protocol. BMI was calculated as measured body weight divided by measured height squared (kg/m^2^).

### Statistical analyses

Data were analysed using SAS 9.4 software (SAS Institute Inc.). Descriptive statistics were used to describe the characteristics of the study population. Cox proportional hazards models were used to estimate the hazard ratios (HR) and 95 % CI for the association of pure fruit juice consumption and fruit consumption with incident CVD, CHD and stroke. Pooled HR were estimated using stratified Cox models, assuming different baseline hazards for the two cohorts. The proportional hazard assumption was fulfilled according to Schoenfeld residuals.

Pure fruit juice consumption was divided into five categories (non-drinkers, <1 glass/week, 1–4 glasses/week, 4–8 glasses/week and ≥8 glasses/week) with the non-drinkers as the reference. Fruit consumption was categorised into quintiles, with the lowest quintile as the reference category. Consideration of potential confounders was based on a difference >10 % in effect estimate between crude and adjusted model and/or theoretical considerations. A total of four statistical models were used to assess the association of pure fruit juice consumption and fruit consumption with CV, CHD and stroke. The first model was adjusted for age and sex. The second model was adjusted for age, sex, educational level, physical activity, smoking, alcohol consumption, DHD15-index and fruit consumption (for associations with pure fruit juice consumption) or pure fruit juice consumption (for associations with fruit consumption). The third and fourth models were additionally adjusted for possible intermediate factors (energy intake, BMI, waist circumference) and important CVD risk factors (systolic blood pressure, TC). In the third model, energy intake and in the fourth model BMI, waist circumference, systolic blood pressure and TC were added as covariates.

Furthermore, we present associations of pure fruit juice with CVD, CHD and stroke for participants with low fruit consumption and high fruit consumption separately to investigate possible benefits of pure fruit juice consumption for people with low fruit consumption. Low fruit consumption was defined as the lowest two quintiles (<121 g/d) and high fruit consumption as the highest three quintiles (≥121 g/d). To formally test the hypothesis of a different effect of pure fruit juice consumption on the risk of CVD, CHD and stroke between low and high fruit consumers, an interaction term was included in the models. The statistical significance level was set at *P* <0·10.

Furthermore, possible effect modification by sex was investigated by including interaction terms in all statistical models. *P* values for linear trend across categories of pure fruit juice consumption and quintiles of fruit consumption were estimated by including fruit and pure fruit juice consumption as continuous variables in the models.

### Sensitivity analyses

Based on the availability of pure and sugar-sweetened fruit juices at the time of assessment, we assume that misreporting (not 100 % pure fruit juice) is unlikely for apple and orange/grapefruit juice consumption but may have occurred for consumption of ‘other fruit juice’. Therefore, a sensitivity analysis was conducted, excluding the sub-item ‘other fruit juice’ for the association with CVD. Furthermore, separate analyses were conducted for apple juice and orange/grapefruit juice consumption with CVD to explore the differences between these types of fruit juices.

## Results

### Descriptive statistics

Of the 34 560 participants, 26·0 % were men and 74·0 % were women (Table 1). The average age at baseline was 48·8 years (sd 11·9 years). Median baseline consumption of fruit and pure fruit juice was 127 g/d (interquartile range = 160 g) and 40 g/d (interquartile range = 119 g), respectively. Of all participants, 1·0 % did not consume fruit and 15·4 % did not consume pure fruit juice at all. Compared with the reference group, pure fruit juice drinkers were more often women, had a higher educational level, were less likely to smoke, more likely to be physically active, were less often heavy alcohol users, had a slightly lower BMI and a lower waist circumference, a lower blood pressure and tended to have healthier dietary habits (Table 1). Similar results were observed for high fruit consumers compared with the reference group except for age (online Supplementary Table S1): high fruit consumers tended to be older than low fruit consumers, while pure fruit juice drinkers tended to be younger than non-drinkers.

**Table 1:** Baseline characteristics according to categories of pure fruit juice consumption (*n* 34 560) (Numbers and percentages; mean values and standard deviations; medians and interquartile ranges (IQR))

			Categories of pure fruit juice consumption									
	All participants		Non-drinkers (*n* 5317)		<1 gl/week^*^ (*n* 7727)		1–4 gl/week^*^ (*n* 9417)		4–8 gl/week^*^ (*n* 9019)		≥8 gl/week^*^ (*n* 3080)	
	%	*n*	%	*n*	%	*n*	%	*n*	%	*n*	%	*n*
Cohort												
Prospect	41·9	14 474	45·9	2441	39·1	3020	38·5	3629	47·4	4278	35·9	1106
MORGEN	58·1	20 086	54·1	2876	4707·0	60·9	61·5	5788	52·6	4741	64·1	1974
Sex												
Male	26·0	8992	30·0	1597	29·7	2291	27·2	2565	19·5	1755	25·5	784
Education level												
Low	56·8	19 617	73·3	3895	53·9	4166	52·6	4951	54·9	4947	53·8	1658
Intermediate	22·3	7720	15·2	809	23·9	1843	24·0	2262	22·8	2052	24·5	754
High	20·9	7223	11·5	613	22·2	1718	23·4	2204	22·4	2020	21·7	668
Smoking status												
Never	38·3	13 235	28·8	1531	36·5	2821	39·8	3748	42·4	3821	42·7	1314
Former	31·1	10 745	32·3	1717	33·2	2562	30·6	2881	30·3	2731	27·7	854
Current	30·6	10 580	38·9	2069	30·3	2344	29·6	2788	27·4	2467	29·6	912
Physical activity												
Inactive (moderately)	31·6	10 911	37·0	1967	31·9	2465	30·1	2838	30·8	2773	28·2	868
Active (moderately)	68·4	23 649	63·0	3350	68·1	5262	69·9	6579	69·3	6246	71·8	2212
Alcohol intake												
Never	0·4	129	1·2	61	0·2	13	0·2	15	0·3	26	0·5	14
<10 g ethanol/d	62·4	21 564	59·4	3158	61·6	4763	62·2	5856	62·9	5662	69·0	2125
10–20 g ethanol/d	16·7	5769	15·3	813	17·3	1338	18·0	1693	16·4	1477	14·6	448
20–30 g ethanol/d	10·0	3447	10·9	579	10·3	794	9·7	914	10·4	941	7·1	219
>30 g ethanol/d	10·6	3651	13·3	706	10·6	819	10·0	939	10·1	913	8·9	274

	Mean	sd	Mean	sd	Mean	sd	Mean	sd	Mean	sd	Mean	sd
Age (years)	48·8	11·9	52·1	10·2	48·2	11·6	47·4	11·8	49·6	12·2	45·9	13·1
BMI (kg/m^2^)	25·5	3·9	26·1	4·1	25·4	3·8	25·4	3·8	25·4	3·8	25·5	4·1
Waist circumference (cm)	84·9	11·3	87·2	11·8	84·8	11·3	84·6	11·2	83·9	10·8	84·6	11·5
Diastolic blood pressure (mmHg)	77·7	10·6	79·2	10·4	77·6	10·4	77·4	10·5	77·5	10·7	77·2	11·1
Systolic blood pressure (mmHg)	125·8	18·6	128·7	19·0	125·1	18·1	124·7	18·1	126·0	19·0	124·8	18·8
Total cholesterol (mmol/l)	5·63	1·15	5·84	1·14	5·59	1·13	5·57	1·15	5·64	1·16	5·52	1·18
HDL-cholesterol (mmol/l)	1·42	0·40	1·39	0·41	1·43	0·40	1·42	0·39	1·45	0·40	1·40	0·38
Total:HDL-cholesterol ratio	4·23	3·60	4·43	8·50	4·20	1·49	4·20	1·49	4·17	1·45	4·23	1·49
DHD15-index	66·1	14·4	62·7	14·6	65·7	14·4	66·5	14·3	68·3	13·9	65·6	14·7

	Median	IQR	Median	IQR	Median	IQR	Median	IQR	Median	IQR	Median	IQR
Fruit consumption (g/d)	127	160	124	183	124	173	125	160	181	166	178	169
Pure fruit juice consumption (g/d)	40	119	0	0	12	14	49	23	131	14	267	34
SSB (g/d)†	45	107	35	105	35	95	49	101	49	105	69	155
Dairy product beverages (g/d)	177	336	93	242	146	262	200	351	200	355	200	348
Total energy intake (kJ/d)	8238	3134	7715	3293	8058	3130	8364	3075	8213	2858	9121	3376
Total energy intake (kcal/d)	1969	749	1844	787	1926	748	1999	735	1963	683	2180	807

MORGEN, Monitoring Project on Chronic Disease Risk Factors; DHD15-index, Dutch Healthy Diet index 2015 (indicator for dietary habits); SSB, sugar-sweetened beverages.

* gl/week = glass(es) of 150 ml/week.

† SSB included Roosvicee/Karvan cevitam, coke and other sugar-containing soft-drinks.

### Associations of pure fruit juice consumption with CVD, CHD and stroke

After an average follow-up of 14·6 years, 3801 CVD events, including 2135 CHD events and 1135 stroke events, had occurred. After adjustment for age, sex, educational level, physical activity, smoking, alcohol consumption, DHD15-index and fruit consumption, compared with no consumption, the lowest three categories of pure fruit juice consumption were significantly associated with a reduced risk of CVD, with HR ranging from 0·85 to 0·88 (Table 2; model 2). The highest category of pure fruit juice consumption (≥8 glasses/week) was not significantly associated with the risk of CVD (HR: 0·97; 95 % CI 0·85, 1·10). For CHD, we found very similar results. Only the lowest three categories of pure fruit juice consumption were significantly associated with HR ranging from 0·83 to 0·86 (Table 2; model 2), but the highest category not. For stroke, only two categories were significantly associated. Consumption of 1–4 glasses per week and 4–8 glasses per week was significantly associated with a lower risk of stroke, with HR of 0·80 (95 % CI 0·64, 0·99) and 0·76 (95 % CI 0·61, 0·94), respectively. The lowest and the highest categories of pure fruit juice consumption were not significantly associated with the risk of stroke. No statistically significant interactions were found between sex and pure fruit juice consumption for CVD, CHD and stroke, with *P* values of 0·74, 0·27 and 0·76, respectively.

**Table 2:** Association between pure fruit juice consumption and CVD, CHD and stroke^*^ among 34 560 European Prospective Investigation into Cancer and Nutrition–Netherlands participants (Hazard ratios (HR) and 95% confidence intervals)

	Categories of pure fruit juice consumption									
	Non-drinkers	<1 gl/week†		1–4 gl/week†		4–8 gl/week†		≥8 gl/week†		*P*_trend_
	HR	HR	95% CI	HR	95% CI	HR	95% CI	HR	95% CI	
*n*	5317	7727		9417		9019		3080		
CVD (*n*)	858	802		916		912		313		
Person years (mean)	14·1	14·5		14·5		14·4		14·4		
Model 1	1·00	0·77	0·70, 0·85	0·76	0·69, 0·84	0·73	0·67, 0·80	0·85	0·75, 0·97	0·10
Model 2	1·00	0·87	0·79, 0·96	0·88	0·80, 0·97	0·85	0·77, 0·94	0·97	0·85, 1·10	0·82
Model 3	1·00	0·88	0·80, 0·97	0·89	0·81, 0·98	0·86	0·78, 0·95	0·98	0·86, 1·12	0·96
Model 4	1·00	0·89	0·81, 0·98	0·89	0·81, 0·97	0·87	0·79, 0·95	0·96	0·84, 1·09	0·67
CHD (*n*)	499	444		518		506		168		
Person years (mean)	14·4	14·7		14·7		14·6		14·6		
Model 1	1·00	0·73	0·64, 0·83	0·74	0·66, 0·84	0·72	0·63, 0·81	0·80	0·67, 0·95	0·17
Model 2	1·00	0·83	0·73, 0·95	0·86	0·76, 0·97	0·84	0·74, 0·95	0·89	0·75, 1·06	0·64
Model 3	1·00	0·83	0·73, 0·95	0·86	0·76, 0·98	0·84	0·74, 0·96	0·90	0·75, 1·07	0·72
Model 4	1·00	0·85	0·75, 0·97	0·87	0·77, 0·98	0·86	0·76, 0·98	0·89	0·74, 1·06	0·59
Stroke (*n*)	179	170		168		177		72		
Person years (mean)	14·8	15·0		14·9		14·8		14·8		
Model 1	1·00	0·81	0·66, 1·00	0·69	0·56, 0·86	0·66	0·53, 0·81	0·97	0·73, 1·27	0·33
Model 2	1·00	0·92	0·74, 1·14	0·80	0·64, 0·99	0·76	0·61, 0·94	1·09	0·83, 1·44	0·72
Model 3	1·00	0·93	0·75, 1·15	0·81	0·65, 1·00	0·77	0·62, 0·96	1·12	0·85, 1·48	0·85
Model 4	1·00	0·93	0·75, 1·15	0·80	0·65, 0·99	0·77	0·62, 0·95	1·08	0·82, 1·42	0·65

DHD15-index, Dutch Healthy Diet index 2015.

* Model 1: adjusted for age and sex. Model 2: adjusted for age, sex, educational level, physical activity, smoking, alcohol consumption, DHD15-index (indicator for dietary habits), fruit consumption. Model 3: adjusted for age, sex, educational level, physical activity, smoking, alcohol consumption, DHD15-index (indicator for dietary habits), fruit consumption, energy intake. Model 4: adjusted for age, sex, educational level, physical activity, smoking, alcohol consumption, DHD15-index (indicator for dietary habits), fruit consumption, BMI, waist circumference, systolic blood pressure and total cholesterol.

† gl/week = glass(es) of 150 ml/week.

### Associations in low and high fruit consumers

Although the associations between pure fruit juice consumption and CVD, CHD and stroke differed slightly for low and high fruit consumers (Table 3), HR were not consistently higher or lower for low and high fruit consumers. Formal testing for interaction also showed that level of fruit consumption did not modify the association of pure fruit juice consumption with CVD (*P* = 0·64), CHD (*P* = 0·70) and stroke (*P* = 0·23).

**Table 3:** Association between pure fruit juice consumption and CVD, CHD and stroke^*^ for low and high fruit consumers (Hazard ratios (HR) and 95% confidence intervals)

	Categories of pure fruit juice consumption									
	Non-drinkers	<1 gl/week†		1–4 gl/week†		4–8 gl/week†		≥8 gl/week†		*P*_trend_
	HR	HR	95% CI	HR	95% CI	HR	95% CI	HR	95% CI	
Participants with low fruit consumption (*n*)	2392	3498		4079		2829		1025		
CVD (*n*)	404	363		391		279		101		
Model 2	1·00	0·84	0·73, 0·97	0·85	0·74, 0·99	0·85	0·73, 1·00	0·94	0·76, 1·18	0·56
CHD (*n*)	225	205		227		160		53		
Model 2	1·00	0·86	0·71, 1·05	0·90	0·74, 1·09	0·91	0·74, 1·12	0·89	0·66, 1·21	0·78
Stroke (*n*)	83	69		64		47		19		
Model 2	1·00	0·84	0·61, 1·16	0·73	0·52, 1·02	0·72	0·50, 1·03	0·91	0·55, 1·51	0·45
Participants with high fruit consumption (*n*)	2925	4229		5338		6190		2055		
CVD (*n*)	454	439		525		633		212		
Model 2	1·00	0·90	0·79, 1·03	0·90	0·80, 1·03	0·87	0·77, 0·99	0·99	0·84, 1·17	0·78
CHD (*n*)	274	239		291		346		115		
Model 2	1·00	0·81	0·68, 0·96	0·83	0·70, 0·98	0·81	0·69, 0·95	0·88	0·70, 1·09	0·73
Stroke (*n*)	96	101		104		130		53		
Model 2	1·00	1·00	0·76, 1·33	0·87	0·66, 1·16	0·82	0·63, 1·07	1·21	0·87, 1·70	0·90

* Model 2: adjusted for age, sex, educational level, physical activity, smoking, alcohol consumption, Dutch Healthy Diet index 2015 (indicator for dietary habits), fruit consumption.

† gl/week = glass(es) of 150 ml/week.

### Associations of fruit consumption with CVD, CHD and stroke

After adjustment for age, sex, educational level, physical activity, smoking, alcohol consumption, DHD15-index and pure fruit juice consumption, compared with the lowest quintile of fruit consumption, the highest three quintiles (121–186, 186–259 and ≥259 g/d) were statistically significantly associated with a lower incidence of CVD, with HR ranging from 0·87 to 0·88 (Table 4; model 2). No statistically significant association was found between fruit consumption and CHD and between fruit consumption and stroke (Table 4; model 2). No statistically significant interactions by sex were observed for the association between fruit consumption and outcomes, with *P* values for interaction for CVD, CHD and stroke of 0·12, 0·78 and 0·34, respectively.

**Table 4:** Association between fruit consumption and CVD, CHD and stroke^*^ among 34 560 European Prospective Investigation into Cancer and Nutrition–Netherlands participants (Hazard ratios (HR) and 95% confidence intervals)

	Quintiles of fruit consumption									
	<69 g/d	69–121 g/d		121–186 g/d		186–259 g/d		≥259 g/d		*P*_trend_
	HR	HR	95% CI	HR	95% CI	HR	95% CI	HR	95% CI	
*n*	6911	6912		6913		6912		6912		
CVD (*n*)	794	744		745		779		739		
Person years (mean)	14·3	14·4		14·4		14·4		14·4		
Model 1	1·00	0·83	0·75, 0·92	0·76	0·68, 0·84	0·74	0·66, 0·82	0·69	0·62, 0·76	<0·0001
Model 2	1·00	0·92	0·83, 1·02	0·87	0·78, 0·97	0·88	0·80, 0·98	0·87	0·78, 0·97	0·10
Model 3	1·00	0·93	0·84, 1·03	0·88	0·79, 0·97	0·89	0·80, 0·99	0·89	0·79, 0·99	0·18
Model 4	1·00	0·92	0·83, 1·02	0·87	0·78, 0·96	0·89	0·80, 0·99	0·87	0·78, 0·97	0·12
CHD (*n*)	451	419		420		444		401		
Person years (mean)	14·5	14·6		14·6		14·6		14·6		
Model 1	1·00	0·86	0·76, 0·99	0·80	0·70, 0·92	0·81	0·70, 0·92	0·73	0·63, 0·84	0·0005
Model 2	1·00	0·95	0·83, 1·09	0·91	0·79, 1·05	0·95	0·83, 1·09	0·91	0·79, 1·06	0·64
Model 3	1·00	0·96	0·83, 1·10	0·91	0·80, 1·05	0·95	0·83, 1·10	0·92	0·79, 1·07	0·74
Model 4	1·00	0·95	0·83, 1·09	0·91	0·79, 1·04	0·95	0·83, 1·09	0·91	0·79, 1·07	0·70
Stroke (*n*)	145	137		152		169		163		
Person years (mean)	14·8	14·9		14·9		14·8		14·9		
Model 1	1·00	0·77	0·61, 0·97	0·75	0·59, 0·94	0·74	0·59, 0·93	0·69	0·55, 0·87	0·0016
Model 2	1·00	0·88	0·69, 1·11	0·88	0·70, 1·12	0·93	0·74, 1·18	0·92	0·72, 1·17	0·32
Model 3	1·00	0·88	0·70, 1·12	0·90	0·71, 1·13	0·95	0·75, 1·20	0·94	0·73, 1·20	0·43
Model 4	1·00	0·89	0·70, 1·12	0·89	0·70, 1·12	0·95	0·75, 1·20	0·93	0·73, 1·19	0·38

DHD15-index, Dutch Healthy Diet index 2015.

* Model 1: adjusted for age and sex. Model 2: adjusted for age, sex, educational level, physical activity, smoking, alcohol consumption, DHD15-index (indicator for dietary habits), pure fruit juice consumption. Model 3: adjusted for age, sex, educational level, physical activity, smoking, alcohol consumption, DHD15-index (indicator for dietary habits), pure fruit juice consumption, energy intake. Model 4: adjusted for age, sex, educational level, physical activity, smoking, alcohol consumption, DHD15-index (indicator for dietary habits), pure fruit juice consumption, BMI, waist circumference, systolic blood pressure, total cholesterol.

### Role of adjustment

Adjustment for possible confounders (model 2) attenuated all associations. Additional inclusion of possible intermediate factors and risk factors (models 3 and 4) yielded very similar HR and 95 % CI in all associations.

### Sensitivity analyses

Excluding the subitem ‘other fruit juice’ for the association with CVD yielded similar results as the analysis that included this category (online Supplementary Table S2). Separate analyses for apple juice and orange/grapefruit juice did not show significant differences (online Supplementary Tables S3 and S4).

## Discussion

This study showed that pure fruit juice consumption of up to 7 glasses/week was associated with a 12–15 % lower risk of CVD and a 14–17 % lower risk of CHD. Furthermore, moderate consumption of pure fruit juice was associated with a 20–24 % lower risk of stroke. The associations with pure fruit juice consumption did not differ considerably between low and high fruit consumers. Fruit consumption was associated with a 12–13 % reduced risk of CVD but not with the incidence of CHD and stroke.

We do not have a clear explanation for the absence of a dose–response relationship between fruit juice consumption and CVD. Even low consumption of pure fruit juice (<1 glass/week) was associated with a lower risk of both CVD and CHD. Pure fruit juice drinkers, even those with a consumption of <1 glass a week, had a healthier lifestyle and healthier dietary patterns than non-drinkers (15 % of the total study population). Although we were able to adjust for many relevant confounders, it is possible that we could not fully adjust for possible differences in health and diet-related factors between pure fruit juice drinkers and non-pure fruit juice drinkers. Therefore, residual confounding cannot be ruled out.

### Strengths and limitations

Important strengths of the present study are the prospective design, large sample size and the long follow-up period. Furthermore, EPIC-NL included both men and women from the general population with detailed follow-up data on cardiovascular end points. A validated self-reported FFQ with separate items for consumption of pure fruit juice and sugar-sweetened beverages was used to measure the dietary intake. This study has some limitations. First, it is possible that participants may have reported non-pure fruit juice consumption as pure fruit juice consumption. This may have led to an overestimation of pure fruit juice consumption. Because it is unlikely that misclassification of pure fruit juice consumption is related to CVD incidence, exposure misclassification is likely to have been non-differential and tends to attenuate the observed association towards the null.

Data on pure fruit juice consumption were collected at baseline and it cannot be ruled out that participants changed their consumption during follow-up. Because consumption levels in our study were collected in 1993–1997, we compared them with the consumption levels from the Dutch National Food Consumption Survey 2007–2010^(^ ^25^ ^)^ to explore generalisability. The median pure fruit juice consumption in the Dutch National Food Consumption Survey 2007–2010 was 40 g/d, which is similar to the consumption in the EPIC-NL cohort at baseline. Furthermore, the level of pure fruit juice consumption in our study was within the range observed in different countries worldwide in 2010^(^ ^26^ ^)^.

### Results of other studies

A total of three other epidemiological studies examined the association between pure fruit juice consumption and CVD^(^ ^5^ ^–^ ^7^ ^)^. In the Nurses’ Health Study and the Health Professional’ Follow-up Study, association between citrus fruits and CVD and stroke was studied^(^ ^5^ ^,^ ^6^ ^)^. While no association was observed for CVD, citrus fruit juice consumption was associated with a reduced stroke risk. We found a significant association between pure fruit juice consumption and reduced risk of CVD. For stroke, we found that not the highest category, but the two middle categories of pure fruit juice consumption were significantly associated with a lower risk of stroke. Explaining this difference is difficult, since Joshipura *et al.* ^(^ ^5^ ^)^ did not report the amount of pure fruit juice consumed per quintile. Another study analysed the association between pure fruit juice (orange and grape) consumption and acute coronary syndrome^(^ ^7^ ^)^, which is the composite of unstable angina pectoris, myocardial infarction and cardiac arrest. A 4 % higher risk was observed for women with a higher pure fruit juice consumption, but associations were adjusted for a limited number of dietary factors and could therefore represent an association with other dietary factors than pure fruit juice consumption.

A large number of randomised controlled trials investigated the association between pure fruit juice consumption and cardiometabolic risk factors for CVD, such as blood pressure and cholesterol. A meta-analysis of nineteen randomised controlled trials^(^ ^17^ ^)^ showed that pure fruit juice consumption lowered diastolic blood pressure by 2·07 mmHg. No significant effects were found on TC, HDL-cholesterol and LDL-cholesterol. However, duration of the trials was short (2 weeks to 3 months) and twelve of the nineteen included trials were classified as low quality.

Similarly to our study, fruit consumption has been associated with a lower incidence of CVD^(^ ^3^ ^,^ ^4^ ^)^. Two meta-analyses of cohort studies showed that fruit consumption was associated with a reduced risk of CHD and stroke^(^ ^1^ ^,^ ^2^ ^)^, while we found no association. In the meta-analysis of Dauchet *et al.* ^(^ ^1^ ^)^ six cohorts were included that reported data on fruit intake and CHD and showed a pooled relative risk (RR) of 0·93 (95 % CI 0·89, 0·96) for each increment of one portion fruit per d. The total number of CHD events was 3446. Dauchet *et al.* ^(^ ^2^ ^)^ included five cohorts that reported data on fruit consumption and stroke and showed a pooled RR of 0·89 (95 % CI 0·85, 0·93) for each increment of one portion fruit per d. The total number of stroke events was 1853. Although not statistically significant, our results went in the same direction as the results from these two meta-analyses. The lower total number of CHD and stroke events in our study (2135 and 1135, respectively) could be a possible explanation of our non-significant findings.

### Interpretation of the present study

It has been suggested that the main underlying mechanisms linking pure fruit juice consumption to a lower risk on CVD included antioxidant effects, improvement of aspects of cardiovascular system, improvement of endothelial function, inhibition of platelet aggregation, anti-inflammation and prevention of hyperhomocysteinaemia^(^ ^27^ ^,^ ^28^ ^)^. These effects were related to polyphenols and vitamins. A specific type of polyphenolic antioxidants are flavonoids that are found in pure fruit juice. Results from observational studies suggest that flavonoids might be associated with reduced risk of CVD and CHD^(^ ^13^ ^–^ ^16^ ^)^. In our study, orange/grapefruit juice was consumed most often (61 % of total pure fruit juice consumption) and especially oranges and grapefruits are rich in flavanones. Although the evidence is limited and inconclusive, human intervention studies suggest hypocholesterolaemic effects of flavanone-rich fruits and could be an explanation for the protective association of pure fruit juice consumption on the risk of CVD^(^ ^29^ ^)^. Our sensitivity analyses showed slightly lower point estimates for orange/grapefruit juice consumption than for apple juice consumption. Although the CI largely overlap, it suggests a somewhat stronger association for CVD with orange/grapefruit juice.

There are also possible adverse effects of pure fruit juice consumption. Pure fruit juice consumption might have less effect on satiety than fruit consumption^(^ ^30^ ^)^, and concerns have been raised about pure fruit juice consumption in relation to overweight development, especially in children^(^ ^31^ ^)^. Two systematic reviews and two meta-analyses assessed the association between pure fruit juice consumption and body weight^(^ ^32^ ^–^ ^35^ ^)^ and found that one portion (240 ml) of pure fruit juice per day is associated with a small amount of weight gain in young children and adults. However, it has been suggested that this weight gain is unlikely to be clinically significant^(^ ^36^ ^)^.

The classification of pure fruit juice in the same category as sugar-containing beverages in the Dutch dietary guidelines of 2015^(^ ^10^ ^)^ is based on the comparable sugar content and the expected unfavourable health effects of sugar intake. In our study, we found no adverse consequences of pure fruit juice consumption for the incidence of CVD. However, median consumption of pure fruit juice in the highest category was 267 ml/d (interquartile range = 122 ml), and our results cannot be extrapolated to higher levels of pure fruit juice consumption.

Based on the limited and inconsistent evidence on the association between pure fruit juice consumption and the risk of CVD, further research is needed before definite conclusions can be drawn whether pure fruit juice is an acceptable alternative for fruit and whether there are benefits of pure fruit juice consumption specific to low fruit consumers. An important condition for further research should be assessing 100 % fruit juice consumption with more certainty (i.e. by giving more examples of pure fruit juices and non-pure fruit juices in the questionnaires). It would also be interesting to analyse data from countries with a higher average consumption of pure fruit juice to get more information about the health consequences of high pure fruit juice consumption. In addition, research on the effect of pure fruit juice consumption on other health outcomes is also needed for a balanced consideration of the health benefits and adverse effects of pure fruit juice consumption. Until then, consumption of whole fruit should be preferred over consumption of pure fruit juice because the evidence on the health benefits of fruit is, in contrast to pure fruit juice, more conclusive.

In conclusion, this study showed that low to moderate pure fruit juice consumption, but not consumption of eight or more glasses per week, was associated with a lower risk of CVD, CHD and stroke and fruit consumption with a lower risk of CVD. Associations with pure fruit juice consumption in participants with low and high fruit consumption did not show additional benefits for those with low fruit consumption.

## Supplementary material

For supplementary material accompanying this paper visit https://doi.org/10.1017/S0007114518003380. click here to view supplementary material
