# Supplementary material for: Pure fruit juice and fruit consumption and the risk of CVD: the European Prospective Investigation into Cancer and Nutrition–Netherlands (EPIC-NL) study
Source: Br J Nutr. 2018 Dec 18;121(3):351–9. doi: 10.1017/S0007114518003380 (PMC6390400; doi:10.1017/S0007114518003380)
Supplement: Supplementary file 1 [file S0007114518003380sup001.docx]

| Supplemental table 1. Baseline characteristics according to quintiles of fruit consumption (n=34,560) | | | | | |  |  |  |  |  |
| --- | --- | --- | --- | --- | --- | --- | --- | --- | --- | --- |
|  |  |  |  |  |  |  |  |  |  |  |
|  | Quintiles of fruit consumption | | | | | | | | | |
|  | < 69 g/day (n=6911) | | 69 - 121 g/day (n=6912) | | 121 - 186 g/day (n=6913) | | 186 - 259 g/day (n=6912) | | ≥ 259 g/day (n=6912) | |
|  | % | n | % | n | % | n | % | n | % | n |
| Cohort |  |  |  |  |  |  |  |  |  |  |
| Prospect | 21.6 | 1489 | 33.9 | 2346 | 42.1 | 2910 | 52.3 | 3615 | 59.5 | 4114 |
| MORGEN | 78.5 | 5422 | 66.1 | 4566 | 57.9 | 4003 | 47.7 | 3297 | 40.5 | 2798 |
| Sex |  |  |  |  |  |  |  |  |  |  |
| *male* | 43.9 | 3036 | 28.3 | 1957 | 25.3 | 1749 | 18.3 | 1267 | 14.2 | 983 |
| Education level |  |  |  |  |  |  |  |  |  |  |
| *Low* | 58.1 | 4018 | 55.7 | 3853 | 55.9 | 3867 | 58.6 | 4053 | 55.4 | 3826 |
| *Intermediate* | 25.3 | 1751 | 23.2 | 1605 | 21.6 | 1496 | 21.3 | 1472 | 20.2 | 1396 |
| *High* | 16.5 | 1142 | 21.0 | 1454 | 22.4 | 1550 | 20.1 | 1387 | 24.5 | 1690 |
| Smoking status |  |  |  |  |  |  |  |  |  |  |
| *never* | 28.9 | 1999 | 36.7 | 2537 | 39.9 | 2756 | 40.8 | 2823 | 45.1 | 3120 |
| *former* | 24.0 | 1659 | 29.0 | 2004 | 31.5 | 2180 | 35.4 | 2445 | 35.6 | 2457 |
| *current* | 47.1 | 3253 | 34.3 | 2371 | 28.6 | 1977 | 23.8 | 1644 | 19.3 | 1335 |
| Physical activity |  |  |  |  |  |  |  |  |  |  |
| *(moderately) inactive* | 36.8 | 2546 | 32.5 | 2249 | 31.1 | 2147 | 30.0 | 2073 | 27.4 | 1896 |
| *(moderately) active* | 63.2 | 4365 | 67.5 | 4663 | 68.9 | 4766 | 70.0 | 4839 | 72.6 | 5016 |
| Alcohol intake |  |  |  |  |  |  |  |  |  |  |
| *never* | 0.5 | 35 | 0.3 | 21 | 0.4 | 25 | 0.4 | 24 | 0.4 | 24 |
| *< 10 gram ethanol/day* | 55.9 | 3861 | 60.8 | 4199 | 62.6 | 4328 | 66.1 | 4568 | 66.7 | 4608 |
| *10-20 gram ethanol/day* | 16.1 | 1113 | 17.7 | 1221 | 17.1 | 1183 | 16.6 | 1144 | 16.0 | 1108 |
| *20-30 gram ethanol/day* | 10.5 | 726 | 10.6 | 731 | 10.4 | 722 | 9.0 | 622 | 9.4 | 646 |
| *> 30 gram ethanol/day* | 17.0 | 1176 | 10.7 | 740 | 9.5 | 655 | 8.0 | 554 | 7.6 | 526 |
|  |  |  |  |  |  |  |  |  |  |  |
|  | Mean | SD | Mean | SD | Mean | SD | Mean | SD | Mean | SD |
|  |  |  |  |  |  |  |  |  |  |  |
| Age (years) | 44.0 | 11.9 | 47.1 | 12.0 | 49.0 | 11.9 | 51.4 | 11.1 | 52.3 | 10.7 |
| BMI | 25.5 | 4.1 | 25.4 | 3.8 | 25.5 | 3.9 | 25.7 | 3.8 | 25.6 | 3.8 |
| Waist circumference | 86.4 | 12.3 | 84.7 | 11.4 | 84.9 | 11.2 | 84.5 | 10.9 | 83.8 | 10.5 |
| Diastolic blood pressure (mm Hg) | 77.4 | 10.7 | 77.2 | 10.6 | 77.8 | 10.6 | 78.0 | 10.4 | 78.1 | 10.5 |
| Systolic blood pressure (mm Hg) | 123.3 | 17.6 | 124.2 | 18.3 | 125.9 | 18.8 | 127.5 | 18.7 | 127.9 | 19.1 |
| Total cholesterol (mmol/l) | 5.46 | 1.12 | 5.55 | 1.16 | 5.62 | 1.17 | 5.75 | 1.13 | 5.78 | 1.15 |
| HDL-cholesterol (mmol/l) | 1.34 | 0.38 | 1.41 | 0.39 | 1.42 | 0.39 | 1.45 | 0.40 | 1.48 | 0.40 |
| Ratio total/HDL cholesterol (mmol/l) | 4.39 | 1.61 | 4.22 | 1.54 | 4.14 | 7.46 | 4.24 | 1.45 | 4.17 | 1.41 |
| DHD15-index | 59.5 | 14 | 65.1 | 13.9 | 66.3 | 13.8 | 68.5 | 13.7 | 71.2 | 13.9 |
|  |  |  |  |  |  |  |  |  |  |  |
|  | Median | IQR | Median | IQR | Median | IQR | Median | IQR | Median | IQR |
|  |  |  |  |  |  |  |  |  |  |  |
| Fruit consumption (g/day) | 35 | 35 | 105 | 31 | 127 | 51 | 240 | 14 | 360 | 114 |
| Fruit juice consumption (g/day) | 26 | 73 | 40 | 107 | 49 | 122 | 53 | 121 | 68 | 116 |
| Sugar-sweetened beverages (SSBs) (g/day)* | 69 | 142 | 53 | 112 | 46 | 104 | 35 | 94 | 35 | 77 |
| Dairy beverages (g/day) | 118 | 276 | 200 | 359 | 183 | 316 | 200 | 358 | 200 | 360 |
| Total energy intake (kcal/day) | 2072 | 869 | 1986 | 769 | 1973 | 748 | 1908 | 694 | 1917 | 657 |
| DHD15-index: Dutch Healty Diet index 2015. *SSB included Roosvicee/Karvan cevitam, coke and other sugar-containing soft-drinks. | | | | | | |  |  |  |  |
